# Prediction model of adult flight activities of *Synanthedon bicingulata* (Lepidoptera, Sesiidae)

**DOI:** 10.1016/j.heliyon.2025.e41856

**Published:** 2025-01-10

**Authors:** Hyeon-Ji Yang, Seonwoong Nah, Hyunjung Kim, Sunghoon Baek

**Affiliations:** a R&D Center, Epinet, Anyang-Si, 14057, Republic of Korea; b Horticulture Division, Jeonbuk State Agricultural Research & Extension Services, Iksan-Si, 54591, Republic of Korea

**Keywords:** *Synanathedon bicingulata*, Phenological model, Degree-day model, Future distribution, Climatic change

## Abstract

Clearwing moth, *Synanthedon bicingulata*, was first reported in 1931 in Korea, but its economic damage has been suddenly increasing with the climatic change. However, a prediction model for the adult occurrence of *S. bicingulata* has not yet been developed. Thus, this study was aimed to develop a forecasting model for adult occurrence and to predict its future occurrence. The historical flight activity data of *S. bicingulata* were fitted with the two-peaked Weibull function against its developmental degree-days with the lower developmental threshold, 4 °C, and the bio-fixed date, October 1 in the previous year. The developed model well predicted its historical emergence in field conditions. When the model was applied to climate change scenarios, it was expected that the future occurrence of *S. bicingulata* could become earlier than the current. Thus, the results of this study should be helpful in managing *S. bicigulata* populations.

## Highlights

• Degree-day model well predicted *S. bicingulata* field emergence.
• Adults of *S. bicingulata* showed two occurrence peaks per a year.
• The future occurrence of *S. bicingulata* could become earlier than the current.
• Developed model aids to manage *S. bicingulata* populations.

## 1 Introduction

Clearwing moths are worldwide distributed polyphagous pests although most occur in the tropics [1,2]. The occurrence of the moth in Korea was first documented by Matsumura in 1931 [2]. Since the first reports, many species have been newly found. Currently, 25 species in nine genera of Sesiidae, Lepidoptera have been formally identified [3].

Among these moths, *Synanthedon bicingulata* has been causing serious economic damage in stone fruits in Korea [4]. Recently, its density and damage increased by attacking Japanese cherry blossom (*Prunus serrulate* var. *spontanea*), a popular roadside tree in Korea [5]. This phenomenon has made worse in the management of *S. bicingulata* in both urban and rural areas, causing more than 60 % damage rates of *Prunus* trees in certain regions of Korea [6].

The adults of *S. bicingulata* lay eggs in the cracks, wounds, and underneath the bark of the lower trunk of trees [7], causing damage mainly around 50 cm above the ground of a tree [5]. The hatched larvae penetrate trunks near the oviposited area, and then they feed on cambium inside trunks. The feeding by a few larvae could break a lot of branches within a contaminated tree, which causes economic damage [8]. Their continuous feeding weakens the contaminated trees resulting in complications of tree growth and eventually death of all branches of the trees [9]. Consequently, serious damage could not be avoided without effective control of *S. bicingulata* at its adult stage which is only exposed growth stages to the surrounding environment [7]. Insecticide applications would be most effective at the adult stage of moth.

Forecasting the adult occurrence of *S. bicingulata* is possible by theoretical models (e.g., integrating temperature-dependent development models) [[10], [11], [12]] or phenological models (e.g., applying a correlative approach to its occurrence in field conditions with degree-days) [[13], [14], [15]]. For *S. bicingulata*, it is difficult to develop a theoretical model because its larvae develop within a tree. However, phenology models of the moth could be developed by using its occurrence data from published studies conducted under field conditions [5,[16], [17], [18], [19]]. Currently, no phenological models for *S, bicingulata* have been developed even if its occurrence data under field conditions are available in Korea. The Rural Development Administration (RDA) of Korea only provided information on the estimated degree-days for two occurrence peaks of *S. bicingulata* without a defined model [20].

To develop a phenology model of *S. bicingulata* adults, the number of its occurrence peak is important to determine a model to be applied. Lee et al. [7] reported that *S. bicingulata* lays eggs only once a year in Korea. However, previous studies [5,[16], [17], [18], [19]] clearly showed two occurrence peaks of its adults. It is necessary therefore to make sure the occurrence peaks of *S. bicingulata* in Korea.

The hatched larvae in tree cracks, wounds, and underneath of bark move into the center of tree through galleries within tree trunks which the larvae bore as they grow [7]. It is known that the larvae of *S. bicingulata* stop their development when they are exposed to unfavorable environmental conditions (i.e., the temperatures under its lower developmental threshold temperature) without obligatory overwintering during winter [7]. When the temperature increases above its lower developmental threshold in the spring of the next year, they resume their development [7]. Due to this ecological characteristic, their occurrence should be affected by temperatures during winter and spring. According to the Korea climate change assessment report for the last 100 years [21], the increase in average temperatures was 0.25 and 0.24 °C per 10 years in the winter and spring, respectively. Moreover, the Intergovernmental Panel on Climate Change (IPCC) reported that the temperature increase during winter and spring would be accelerated in the future according to the shared socioeconomic pathway (SSP) scenarios. Thus, the occurrence pattern of *S. bicingulata* adults would be changed in the case of climate change in the future.

In this study, we developed a forecasting model for the adult occurrence of *S. bicingulata*. The developed model was used to predict the change of the occurrence patterns for the future according to the SSP scenarios. Based on the results, we discussed voltinism and the future occurrence of *S. bicingulata* in Korea.

## 2 Material & methods

### 2.1 Adult moth occurrence data

The occurrence data of *S. bicingulata* adults were obtained from published papers [5,[16], [17], [18], [19]] (Table 1). The host of each study was *Prunus serrulate* var. *spontanea* [5], *P. mume* [16], *P. persica* [17], *Diospyros kaki* var. ‘Dangam’ [18], and *Ziziphus jujuba* var. ‘Inermis’ [19]. In each figure, the sampling date (*x*-axis) and caught number (*y*-axis) for individual sampling data were traced using WebPlotDigitizer [22]. Twenty-seven data sets were obtained from the five papers [5,[16], [17], [18], [19]]. Among them, 24 data sets were used to develop a forecast model predicting the occurrence of adults based on daily average temperature. The model validation was carried out using the other three data sets, in which the early occurrence of *S. bicingulata* was not observed and the observation years were different among the selected data sets.

**Table 1:** Experimental plot locations, observation periods, and traps and sex-pheromones used in the five studies [5,[16], [17], [18], [19]].

Ref.	Location		Observation		Trap		Sex-Pheromone
	Name	Geocoordinate (lat./long.)	Year	Data Point (number^a^)	Shape	Manu-facturer	
16	Hwasim-ri	35.0932/127.7224	2007	17	Cylindrical and delta Sticky trap	GA^b^	GA
	Meokjeomgol	35.1230/127.7226	2005	14			
			2006	17			
			2007	17			
			2005	20			
			2006	29			
17	Suwon-si	37.2663/126.9740	2009	18	Delta	GA	GA
			2010	18			
			2011	18			
18	Changwon-si	35.1558/128.5794	2013	32	Cylindrical or delta	GA	GA
			2014	27			
	Jinju-si (A)	35.1413/128.1367	2014	25			
			2015	30			
	Suncheon-si	34.9285/127.4557	2014	25			
			2015	30			
	Munsan-eup	35.1604/128.1692	2015	30			
19	Boeun-gun	36.4900/127.7293	2013	13	Delta	GA	GA
			2014	13			
			2015	13			
5	Gongju-si	36.3583/127.2478	2017	13	Bucket	KIP^c^	KIP
			2018	13			
	Jinju-si (B)	35.1996/128.1726	2017	13			
			2018	13			
	Chuncheon-si	37.9285/127.7743	2017	13			
			2018	13			
	Seoul	37.4583/126.9564	2017	13			
			2018	13			

a Number indicates the number of data points of each dataset.

b Green Agrotech Co., Ltd. (Gyeongsan, Korea).

c KeiAiPi (KIP) (Daejeon, Korea).

### 2.2 Accumulation of degree-days

The cumulative degree-days (CDDs) for the occurrence of *S. bicingulata* adults were calculated using the single-sine method [23]. To accumulate degree-days required to complete *S. bicingulata* individuals’ development, temperature data (i.e., daily maximum and minimum air temperatures), the lower developmental threshold (LDT) of its development, and the biofix timing, at which the degree-day accumulation was started, were required. The Korea Meteorological Administration (KMA) provided daily maximum and minimum ambient temperature data which were collected from the automated weather stations installed at the nearest sites from the experimental plots. The distances between the observation site and the weather station were 8.5, 9.5, 1.2, 1.7, 9.1, 12.9, 11.8, 0.5, 5.1, 12.7, 2.7, and 0.8 km at Hwasim-ri, Meokjeomgol, Suwon-si, Changwon-si, Jinju-si (A), Sucheon-si, Munsan-eup, Boeun-gun, Gongju-si, Jinju-si (B), Chuncheon-si, and Seoul observation site, respectively. The LDT applied in the present study was 4 °C, which was determined by Bergh et al. [24] for *S. scitula*, dogwood borer. Similar to *S. bicingulata*, *S. scitula* overwinters as the larval stage inside of tree trunk without the obligatory overwintering. The biofix timing date at which the degree-day accumulation was started was October 1st in the present study. Bergh et al. [24] found that accumulation of degree-days from October 1st resulted in a better forecast of the adult occurrence of *S. scitula* in the next year than the biofix timing of January 1st, which was commonly used for insect forecast models [[10], [11], [12], [13], [14], [15]].

### 2.3 Model development

For each site, the adult occurrence was transformed into the cumulative proportion (%) by dividing the accumulated trapped adult number by the total number of adults per year. The cumulative proportions were fitted with a two-peak Weibull function [25] against the calculated CDDs:

$$P\left({DD}_{x}\right)=\frac{\alpha_{1}}{1+{\left(\frac{{DD}_{x}}{\beta_{1}}\right)}^{\gamma_{1}}}+\frac{\alpha_{2}}{1+\exp\;\left[-\frac{{DD}_{x}-\left(\beta_{1}+\Delta \beta \right)}{\gamma_{2}}\right]}$$

where *P*(*DD*_*x*_) is the cumulative proportion of trapped adults at degree days (*DD*_*x*_), *α*_1_ and *α*_2_ are the relative proportions of two adult occurrence peak (*α*_1_ + *α*_2_ = 1), *β*_1_ is the CDD at 50 % adult occurrence at the first peak, Δ*β* is the CDD between the first and second 50 % occurrence time, and *γ*_1_ and *γ*_2_ are the steepness of the non-linear lines describing first and second peak, respectively. The Weibull function parameters were estimated using PROC NLIN of SAS (SAS Institute Inc.; Cary, NC, USA) [26].

### 2.4 Model validation

Model validation was conducted using three data sets from Suncheon-si in 2015, Chucheon-si in 2017, and Gongju-si in 2018 (Table 1). The CDDs for each of the three sites were calculated with the single-sine method [23] with LDT 4.0 °C [24] and the biofix of October 1st [24]. For each site, adult occurrences were compared at 10, 30, 50, 70, and 90 % cumulative occurrences estimated from the nearest two points around a target cumulative occurrence through the linear regression. For comparison, the CDD values at each cumulative occurrence proportion were transformed as the Julian days.

Moreover, the results of the developed models in this study were also compared with the CDDs (i.e., 350 DDs for the first peak and 1895 DDs for the second peak), of two peak timing of *S. bicingulata* adult occurrence suggested in the RDA [20] in Korea. Three data sets used in validation were used again. To estimate the Julian days based on the RDA suggestion [20], the used LDT and bio-fixed timing were 8.1 °C and January first, respectively. The CDDs for the RDA suggestion [20] were also calculated with the single-sine method [23].

### 2.5 Application to the past and future climate changes

Maps of daily mean maximum and minimum ambient temperatures maps were downloaded for 2010s, 2030s, 2070s, and 2100s from the website of KMA database (http://www.climate.go.kr). The present study used both SSP 1–2.6 and SSP 5–8.5 scenarios of the future temperature because these scenarios represented extreme conditions among four scenarios. In order to generate the climate maps of daily maximum and minimum ambient temperature for the last 30 years, the spatial interpolation with the inverse distance weight method and the topo-climatology model [27] were applied using point temperature data downloaded from the KMA database. The CDDs were calculated from October 1st to thermal constant (DDs) of *S. bicingulata* second peak using the LDT 4 °C with the single sine method [23] using the Python version 3.10 (Python Software Foundation; Wilmington, DE, USA). The estimated first and second occurrence peaks of *S. bicingulata* were visualized with QGIS version 3.16.9 [28].

## 3 Results

**Fig. 1:**
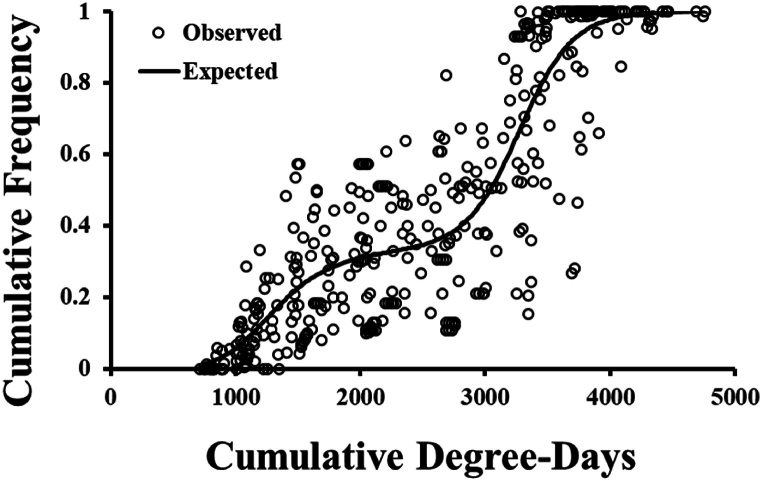
Cumulative observed and predicted occurrence of *S. bicingulata* adults in field conditions.

The occurrence of *S. bicingulata* adults clearly showed two peaks in all data sets (Fig. 1). The developed two-peak model could explain well the variations in adult occurrence of *S. bicingulata* (Table 2; *F* = 495.4, df = 4, 432, *P* < 0.001). The first and second peak occupied approximately 40 and 60 %, respectively, of the numbers among total trapped numbers during a crop season (Table 2). The estimated CDDs of each occurrence peak were 1464.1 and 3293.3 DDs for the first and second peaks, respectively (Fig. 1, Table 2).

**Table 2:** Estimated parameters and SEM of each parameter in the developed two-peak Weibull model.

Parameters	$\alpha_{1}$	$\beta_{1}$	$\gamma_{1}$	$\alpha_{2}\left(1-\alpha_{2}\right)$	Δβ	$\gamma_{2}$
Estimates	0.4008	1464.09	−4.9041	0.5992	1829.17	189.73
SEM	0.0415	92.95	1.1323	–	78.28	28.75

Even though there were variations according to the regions, the deviations between observed and expected dates became smaller from the early cumulative occurrence (i.e., 10 %) to the peak occurrence (i.e., 50 %), and larger from the peak occurrence to the late occurrence (i.e., 90 %) in both peaks (Table 3). The average deviations were about 6.3 and 4.0 days for the first and second occurrence peaks, respectively (Table 3). This prediction accuracy was much better than the one [20] suggested by the RDA in Korea (Table 4). This study decreased approximately 3.0 and 5.6 days in deviations for the first and second occurrence peaks, respectively (Table 4).

**Table 3:** Comparisons between observed and predicted occurrence (observed/predicted Julian date) of *S. bicingulata* adults at 10, 30, 50, 70, and 90 % occurrence time.

Peak	Cumulative occurrence (%)^a^	Suncheon	Chuncheon	Gongju	Avg. deviation ± SD
First peak	10	109/111	130/124	134/128	4.7 ± 2.31
	30	122/133	138/144	149/148	6.0 ± 5.00
	50	137/146	149/158	160/159	6.3 ± 4.62
	70	152/159	163/171	167/171	6.3 ± 2.08
	90	162/174	178/187	176/184	9.7 ± 2.08
Second peak	10	240/215	244/222	232/219	20.0 ± 6.24
	30	243/234	248/236	245/237	9.7 ± 2.08
	50	244/243	251/245	251/246	4.0 ± 2.65
	70	245/252	255/255	259/256	3.3 ± 3.51
	90	245/269	265/271	273/274	10.3 ± 12.10

a Each cumulative occurrence was estimated from the nearest two points around a target occurrence through the linear regression.

**Table 4:** Differences (days) between model predictions and observed data at two occurrence peaks of *S. bicingulata* adults in two studies.

Locations	This study		RDA (2000)	
	First peak	Second peak	First peak	Second peak
Suncheon-si	9	1	2	1
Chuncheon-si	9	6	9	15
Gongju-si	1	5	17	13

Average (Mean ± SD)	6.3 ± 4.62	4.0 ± 2.65	9.3 ± 7.51	9.6 ± 7.57

**Fig. 2:**
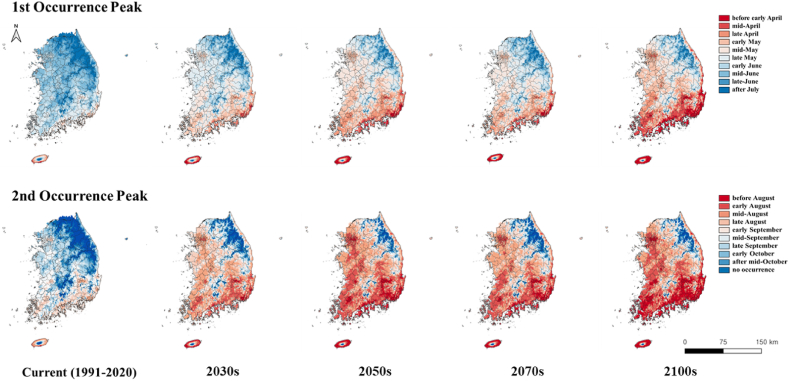
Predicted 50 % occurrence timing of *S. bicingulata* adults based on daily maximum and minimum temperature data of the current (1991–2020) and futures (2030s, 2050s, 2070s, and 2100s) based on SSP 1–2.6 scenarios.

**Fig. 3:**
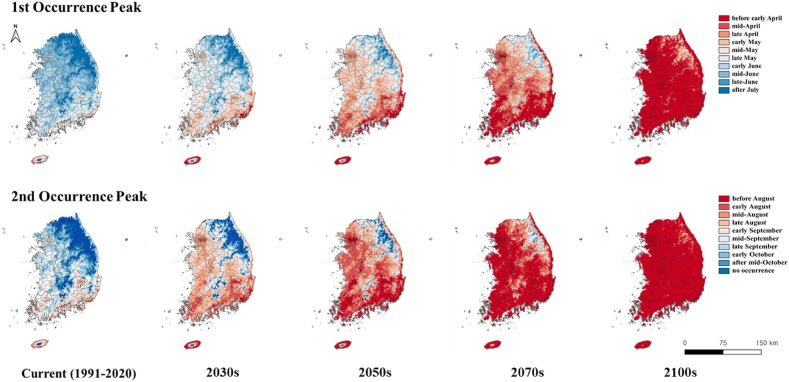
Predicted 50 % occurrence timing of *S. bicingulata* adults based on daily maximum and minimum temperature data of the current (1991–2020) and futures (2030s, 2050s, 2070s, and 2100s) based on SSP 5–8.5 scenarios.

The forecasting maps of the first and second occurrences of *S. bicingulata* adults based on the average daily min- and max-temperatures during the 30 years (1991–2020) indicated that the first and second occurrence peaks would occur between the late of May and the middle of June and between the late of August and the middle of September, respectively (Fig. 2, Fig. 3). As time goes by, these occurrence peaks would be earlier than the one of current regardless of SSP scenarios (Fig. 2, Fig. 3). Especially, it is expected that the changes would be faster for SSP scenario 5–8.5 than for SSP scenario 1–26. The results for the 2100s under the SSP 1–26 scenario were similar to the ones for the 2050s under the SSP 5–8.5 scenario (Fig. 2, Fig. 3).

## 4 Discussion

Temperature played a pivotal role in determining the phenology of *S. bicingulata* adults as other insects [29]. The forecasting model of *S. bicingulata* adults developed in this study also explained variations of their occurrence well and showed good accuracy in predicting their phenology in field conditions. These results indicated that this model could be applied to predict their phenology in the future based on a climatic change scenario.

To develop a phenology model of an insect, its voltinism is required to separate the observed data according to each peak and determine an applied model. In the past, *S. bicingulata* was known that they laid eggs only one time a year in Korea [7]. However, the data used in this study showed that the adults of *S. bicingulata* appeared to have two occurrence peaks per year. Recent studies [5,[16], [17], [18], [19], [20]] found that *S. bicingulata* adults laid eggs two times a year in Korea: The first emergence of adults from overwintered larvae lay eggs from mid-May to mid-June, and the second generation of adults developed from the eggs of the first generation lay eggs from late-August to mid-September in Korea.

This phenology of *S. bicingulata* in Korea was also supported by the required thermal requirements. The CDDs of the first and second peak of *S. bicingulata* adults in this study were expected to be 1464.1 and 3293.6 DDs, respectively. The interval between the first and second peaks was estimated as 1829.2 DDs in this study. This thermal requirement between the first and second peaks was slightly higher than one (i.e., 1150 DDs at LDT 4 °C [30]) required for *S. scitula* development from eggs to adults. By considering thermal requirements for the ovary development, ovipositional peak, and egg development, the thermal requirement of *S. bicingulata* between the first and second peaks might be similar to one of its allied species, *S. scitula.* Moreover, the developmental stage of overwintering *S. bicingulata* larvae was expected as the early developmental stage (e.g., second or third instars) by considering CDDs (i.e., about 365 DDs, extracted the CDDs (1464.1 DDs) in the first adult occurrence peak from the thermal requirement (1829.2 DDs) between the first and second peaks. Kown et al. [31] reported that the developmental stages of *S. bicingulata* of overwintering larvae were the second or third instars.

Even though the developed model in this study was theoretically reasonable, the model prediction resulted in deviations (one to nine days) between predicted and observed days in the peak occurrences of *S. bicingulata* adults depending on locations. This deviation (e.g., seven days) might be inevitable in the insect forecasting models because the temperature data provided by the meteorological administration could not completely reflect the temperatures applied to actual insects in agricultural farms [32]. Thus, the differences between predicted and actual occurrence timings in an insect species could decrease by using micro-environmental data. However, that should require a lot of time, effort, and cost. It could be more reasonable to use a sex-pheromone trap for *S. bicingulata* in each farm level, if more accurate (less than seven days) prediction is demanded. A maximum of two weeks from the expected date in the developed model would be enough to install the traps to monitor the adult flight activities of *S. bicingulata.*

The developed model in this study expected the trapped number of *S. bicingulata* adults at the second peak was higher than at the first peak. It is general in insect phenology because a lot of individuals cannot survive during winter and successfully overwintered individuals lay many eggs during summer [12,13]. The model in this study expected approximately 40 and 60 % among total trapped numbers for the first and second occurrences. Similar ratios have been reported previously [5,16,18,19]. However, Yang et al. [17] found that the proportion of second occurrence peak was extraordinarily high and the ratio between the first and second occurrence was 25 and 75 % in a peach orchard. Differences in the ratio may be due to the host crops and management strategies to control *S. bicingulata*. In Korea, pesticides are highly restricted by law during the fruit ripening to harvesting stages. After fruit harvest, no more applications of pesticides are commonly made until the next crop season. The first and second occurrences of *S. bicingulata* adults were exactly matched with the highly restricted period and no control period in peaches in Korea. These agricultural practices in peaches might affect the high proportion of its second flight activity peak. In most crops and natural conditions, the ratio of the first and second occurrences of *S. bicingulata* adults might be around 40 and 60 %, respectively.

The future distribution of *S. bicingulata* adults based on the climatic change scenarios, SSP scenarios, showed *S. bicingulata* adults would emerge earlier compared to the current. This phenomenon would occur as expected. Especially, SSP 5–8.5 scenario predicted that the first occurrence peak of *S. bicingulata* would occur as early as early February in the 2100s. However, this phenomenon would be difficult to occur. Frank [30] found that *S. scitula*, an allied species of *S. bicingulata*, showed delayed pupa emergence from larva under at 24 °C or 28 °C. They speculated that this developmental delay might be caused by the decreased day length. It was known that *S. scitula* overwintered as the larval stage like *S. bicingulata* [4,5,7,[16], [17], [18], [19], [20],^30^]. This phenomenon indicates that only the larval stage might be able to endure low temperatures during winter. Thus, decreasing the day length would prevent its developmental completion to pupa in *S. bicingulata* until the vernal equinox day. If this phenomenon occurs, the first flight activity peak might be difficult to occur one month earlier than the current even though the climatic change continues as expected.

## 5 Conclusions

In summary, this study provided a forecasting model of *S. bicingulata* flight activities in field conditions. The developed model could be applied to diverse crops with high prediction accuracy. Moreover, this model was theoretically explained. If the climatic change continues, its occurrence will become earlier as time goes by. This information should be helpful in determining its sampling timing and managing *S. bicingulata* populations.

## CRediT authorship contribution statement

**Hyeon-Ji Yang:** Writing – review & editing, Writing – original draft, Visualization, Validation, Software, Methodology, Formal analysis, Data curation, Conceptualization. **Seonwoong Nah:** Writing – review & editing, Visualization, Formal analysis, Data curation. **Hyunjung Kim:** Writing – review & editing, Methodology, Formal analysis, Conceptualization. **Sunghoon Baek:** Writing – review & editing, Writing – original draft, Visualization, Validation, Supervision, Methodology, Formal analysis, Data curation, Conceptualization.

## Data availability statement

The data presented in this study are available upon request from the corresponding author. The data are not publicly available due to institutional policy.

## Additional information

No additional information is available for this paper.

## Ethics declaration

Review and/or approval by an ethics committee as well as informed consent was not required for this study because this article did not involve any direct experimentation/studies on living beings.

## Declaration of competing interest

The authors declare the following financial interests/personal relationships which may be considered as potential competing interests:Hyeon-Ji Yang reports financial support was provided by 10.13039/501100003627Rural Development Administration, Korea. If there are other authors, they declare that they have no known competing financial interests or personal relationships that could have appeared to influence the work reported in this paper.
